# Impact of Cardiovascular Interventions on the Quality of Life in the Elderly

**DOI:** 10.5935/1678-9741.20150080

**Published:** 2015

**Authors:** José Albuquerque de Figueiredo Neto, Lívia Mariane Castelo Branco Reis, Márcia Rodrigues Veras, Lorena Lauren Chaves Queiroz, Karine de Paiva Lima Nogueira Nunes, Priscylla de Oliveira Miranda, Alexsandro Ferreira dos Santos, Joana Kátya Veras Rodrigues Sampaio Nunes

**Affiliations:** 1 Universidade de São Paulo (USP), São Paulo, SP, Brazil; 2 Universidade Federal do Maranhão (UFMA), São Luís, MA, Brazil; 3 Universidade Ceuma, São Luís, MA, Brazil

**Keywords:** Quality of Life, Aged, Cardiovascular Surgical Procedures, Angioplasty, Balloon, Coronary

## Abstract

**INTRODUCTION:**

The elderly population is growing rapidly. Political and socio-economic changes led to the demographic transition in this population with the highest number of surgeries and as well as many comorbidities.

**OBJECTIVE:**

To evaluate the impact of cardiovascular intervention on quality of life of elderly patients after three and six months.

**METHODS:**

Analytical prospective cohort study with elderly between 60 and 80 years of age, of both sexes, with a diagnosis of coronary artery disease and underwent cardiovascular intervention during the period June 2010 to June 2011. Data were collected by individual interviews in the pre and postoperative periods (after three and six months) by telephone. We used the SF-36 to analyse quality of life in order to assess the physical and mental health of the study population.

**RESULTS:**

Of the 44 individuals evaluated, 59.1% were men, 75% in the range of 65 to 74 years, 38.6% were white and 38.6% were black, 31.8% were uneducated, 43.2% were married and 68.2% had less than a minimum wage. Prevailed patients: non-diabetics (68.2%), non-obese (81.8%), hypertensive (84.1%), non-alcoholic and non-smokers (68.2% and 61.4%, respectively). A significant increase in the average of the SF-36 scores between pre and post-surgical periods (three and six months) for the domains: functional capacity, pain, general health, vitality and emotional aspect.

**CONCLUSION:**

The elderly population undergoing intervention may have cardiovascular benefits and improvements of quality of life. Physical fitness improvement measures can be taken to resume that capability.

**Table:** 

**Abbreviations, acronyms & symbols**	
ACTP	= Coronary angioplasty
AMI	= Acute myocardial infarction
CABG	= Coronary artery bypass grafting
CAD	= Coronary artery disease
CVD	= Cardiovascular disease
HF	= Heart failure
NHP	= Nottingham Health Profile
PTCA	= Percutaneous transluminal coronary angioplasty
QOL	= Quality of life
SPSS	= Statistical Package for the Social Sciences

## INTRODUCTION

Population aging in Brazil started from the sixties, twentieth century, when it began to decrease fertility and infant mortality^[1]^. The elderly population grew rapidly, increasing from 8.6% to 11% in ten years^[2]^.

Political and socio-economic changes generated this process of demographic transition. Increased longevity results from the transformation of mortality in children and infectious diseases to increased mortality in the elderly population by chronic and external diseases^[3]^.

Cardiovascular disease (CVD) remains the leading cause of death and disability in the elderly, even with the reduction of smoking and greater access to primary care^[2,4]^.

This population presents the largest number of surgeries and comorbidities. The most performed cardiovascular interventions in the elderly are the myocardial revascularization (CABG) and coronary angioplasty (ACTP), in the treatment of coronary artery disease (CAD), where clogged arteries can cause the acute myocardial infarction (AMI). The risk of complications after CABG and ACTP has been researched in this population, mechanisms were also proposed to reduce morbidity and mortality and improve quality of life (QOL)^[5]^.

Heart failure (HF) comes from physical and psychological symptoms, and is noted as CAD outcome, once installed, have severe impact on the patients' lives^[3]^. The IC is recognized as a public health problem due to its increase each year, and yet, it represents higher costs to social security programs such as licenses and retirements^[6,7]^.

About 49% of CAD deaths occur in developing countries. In developing countries, one third (34%) of the deaths are associated with CAD in 2020. In Northeastern Brazil, the relevance of these causes is also notable, since about 45% of the elderly deaths are due to causes associated with CAD^[8]^.

Although the aging process is not related to coronary heart disease, chronic degenerative diseases are more associated with age, as being quite described in the elderly. And the number of chronic diseases is directly associated with greater functional disability^[9]^.

Cardiovascular diseases compromise QOL of patients by deterioration of cardiac function, as an essential body for maintaining life^[10]^.

Advances in medical and surgical treatment CAD allowed increase in patient survival. With this advance, there are new drugs and procedures that allow the improvement in patients' QOL^[3]^.

To evaluate QOL, the most widely used instrument is the SF-36 (The Medical Outcomes Study 36-item Short-Form Health Survey), being applied in a wide variety of populations, including aspects such as function, dysfunction and emotional well-being and physical condition^[4]^.

Thus, the study sought to assess the impact of cardiovascular surgery on the QOL of elderly patients after three and six months.

## METHODS

Analytical prospective cohort study, conducted at the Federal University of Maranhão - Presidente Dutra Hospital in São Luís, MA, Brazil, approved by the Research Ethics Committee of the institution under protocol number: 005 311/2009-0.

They participated in the study, aged between 60 to 80 years (both sexes), diagnosed with CAD, which were referred for elective cardiovascular interventions of CABG or percutaneous transluminal coronary angioplasty (PTCA), and those who were aware of the Informed Consent. Non-elderly patients with antidepressant drug use or phychiatric disorders were referred for psychotherapy, which harms the understanding and communication in the interview and even patients with acute coronary syndrome requiring emergency surgery or those with compromised ventricular function.

They participated in the cohort study from June 2010 to June 2011, undergoing CABG or ACTP surgery. There were two exclusions from the sample, referring to patients who progressed to death after three months of CABG.

Data were collected by individual telephone interviews conducted before surgery and in the 3^rd^ and 6^th^ month after the surgery.

The validated and generic SF-36 questionnaire was used to assess QOL. The effectiveness of this device in patients undergoing cardiovascular surgery has been reported in the literature^[11]^, the questionnaire can be applied to patients from 12 years of age. Our goal was to assess the physical and mental health conditions.

The SF-36 is multidimensional with eight domains divided into two major components: a) physical component b) the mental component, with a total of 35 questions. The questionnaire also includes an item for evaluation of health changes a year ago (question two), as is noted in Table 1 ^[11]^.

**Table 1:** Component SF-36.

SF-36 Component	Physical	Mental
Domains	Functional capacity (10 items)	Vitality (4 items)
	Physical appearance (4 items)	Social aspect (2 items)
	Pain (2 items)	Emotional aspect (3 items)
	General health (5 items)	Mental health (5 items)
Subtotal of items	21	14
Evaluation of health changes one year ago (1 item)		
Total items	36	

The first data collection was carried out during the week (morning, afternoon or evening). This step took place before the surgical procedure and included conducting interviews to evaluate the clinical and sociodemographic profile, and then, SF-36 questionnaire to assess QOL was applied. In the second stage, three and six months after the intervention, the SF-36 questionnaire was applied again by phone. A unique score for each question was used to interpret the results and converted to a scale of 0 to 100, where values close to zero reflect poor health perception, and numerical scores close to 100 show good health awareness^[12]^.

It is noteworthy that the SF-36 was previously validated for use either through phone calls, interpersonal interviews or under self-administered form, thus no differences were noted between the results obtained^[11,13]^.

We collected the following variables: sex; age (complete years); ethnic group (white, brown and black); origin (São Luís region and other cities in the state of Maranhão); marital status (single, married, common-law marriage, separated, widowed); education (years of schooling completed); and family income (in minimum wages), all self-declared. It also investigated the presence of smoking and drinking.

Data were analyzed using Statistical Package for the Social Sciences (SPSS) for Windows. Descriptive statistical analyzes were performed (frequency tables), mean, standard deviation and median of parametric variables and range of nonparametric variables. Later, the SF-36 was evaluated using the Friedman test. The evaluation of the effect of CABG or PTCA in relation to the domains of the SF-36 was performed using the nonparametric Mann-Whitney test. The evaluation of current health compared a year ago was performed using the chi-square test. Normality was assessed by the Shapiro-Wilk test. The significance level used in the study was 5%.

## RESULTS

Among the 44 patients evaluated, it was noted a frequency of 59.1% (n=26) men, most concentrated in the age group from 65 to 74 years, 75% (n=33), white and brown races were the most prevalent with 38.6% (n=17) each, uneducated individuals were more expressive, 31.8% (n=14). Regarding marital status, 43.2% (n=19) were married and 68.2% (n=30) reported incomes below the minimum wage, concerning the cardiovascular procedure, CABG was carried out in 27.3% (n=12) and PTCA in 72.7% (n=32) (Table 2).

**Table 2:** Distribution of sociodemographic variables in patients undergoing coronary artery bypass grafting or coronary angioplasty, University Hospital President Dutra- HUUFMA. São Luís, MA, Brazil. 2011.

Sociodemographic variables		n	%
Sex	Male	26	59.1
	Female	18	40.9
Age (years)	65-74	33	75.0
	74-85	11	25.0
Ethnic group	White	17	38.6
	Brown	17	38.6
	Black	10	22.7
Education	Unschooled	14	31.8
	Literate	12	27.3
	Fundamental	9	20.5
	Average	5	11.4
	Higher	4	9.1
Marital status	Single	9	20.5
	Married	19	43.2
	Stable union	6	13.6
	Widower	10	22.7
Income	<1 salary	30	68.2
	1 to 2 wages	10	22.6
	2-3 wages	2	4.6
	3 to 4 wages	21	4.6
Cardiac procedure	CABG	12	27.3
	PTCA	32	72.7
Total		44	100.0

CABG=coronary artery bypass grafting; PTCA=percutaneous transluminal coronary angioplasty

Among the prevailed patients, clinical conditions and living habits were evaluated: non-diabetics, 68.2% (n=30), not obese, 81.8% (n=31), hypertensive, 84.1% (n=37), non-alcoholic, 68.2% (n=30) and smokers, 61.4% (n=27), as shown in Table 3.

**Table 3:** Distribution of clinical conditions and habits of life of patients undergoing coronary artery bypass grafting or coronary angioplasty, University Hospital President Dutra-HUUFMA. São Luís, MA, Brazil, 2011.

Variables	N	%
DM		
Yes	14	31.8
Not	30	68.2
Obese		
Yes	8	18.2
Not	31	81.8
HAS		
Yes	37	84.1
Not	7	15.9
Alcoholism		
Yes	14	31.8
Not	30	68.2
Smoking		
Yes	27	61.4
Not	17	38.6
Total	44	100.0

DM=diabetes mellitus; Obese=BMI ≥ 30.0 kg/m^2^; HAS=hypertension

Table 4 showed a statistically significant increase (*P*<0.05) the average of the SF-36 scores between pre and post-surgical periods (three and six months) for the domains: functional capacity, pain, general health, vitality and emotional aspect. The social domain reduced by changing from six months after surgery. The median physical domain did not differ during the follow-up (*P*<0.05).

**Table 4:** Average of the SF-36, preoperatively, and after three and six months of the elderly undergoing coronary artery bypass grafting or coronary angioplasty, University Hospital President Dutra- HUUFMA. São Luís, MA, Brazil, 2011.

Variable	Average	DP	*P*
SF-36			
CF Pre	44.7	27.6	0.0001
CF3	59.3	28.9	
CF 6	60.3	29.1	
FIS Pre*	0	0-100	0.0352
FIS 3*	0	0-100	
FIS 6*	0	0-100	
Pre PAIN	58.7	34.1	0.0001
DOR 3	56.6	26.5	
PAIN 6	62.8	29.8	
HEALTHY Pre	60.9	18.4	0.0265
HEALTHY 3*	57	Nov-92	
HEALTHY 6*	62	Dec-87	
VITAL Pre	59.5	16.2	0.0011
VITAL 3	60.3	17.7	
VITAL 6	62.0	18.1	
SOCIAL Pre*	87.5	12.5-100	0.0047
SOCIAL 3	61.6	18.3	
SOCIAL 6*	75.0	12.5 to 100	
Pre EMO	39.4	43.9	0.0077
EMO 3	43.5	45.3	
EMO 6	44.2	46.4	
MENTAL Pre*	72.0	0-100	0.0520
MENTAL 3	65.8	16.0	
MENTAL 6	64.7	17.2	

CF=functional capacity; FIS=physical appearance; DOR=pain; HEALTHY=General State of Health; VITAL=vitality; SOCIAL=social aspect; EMO=emotional appearance; MENTAL=mental health;

* Non-parametric=median and range

## DISCUSSION

This research, as well as the Armendaris & Monteiro^[14]^ found higher prevalence of women without education or with limited schooling among elderly patients undergoing cardiac surgery. These authors also reported a high frequency of dyslipidemia and hypertension as chronic diseases, agreeing to share with our findings, where the number of hypertensive and diabetic patients was significant. Regarding the frequency of obesity among the evaluated individuals, the prevalence was similar (12%) to the findings.

Christmann et al.^[6]^, when assessing elderly cardiac patients, found higher frequency of men, a finding similar to this research, but also more often of elderly people with low education, 92.5%, and married, 39.2%.

Among the domains of SF-36, there has been a significant improvement in the functional capacity domain (44.7 points *versus* 60.3 points in the preoperative period and six months after surgery) compared to physical, pain, health, vitality and emotional (difference to five points), leaving the caveat that all areas have remained above 50^[6]^ points out that the low QOL in this age group in association with heart disease can lead to serious commitments.

It is not new that cardiovascular surgery can positively affect the QOL of elderly patients^[6]^. However, defining the impact of this event on QOL can be an indispensable task to carry out psychological interventions in the pre, peri and post-surgical period, aggravating to design ever smaller, in activities of daily living, functional and mental capacity of this population.

Santana et al.^[15]^, in their research in Aracaju, Sergipe, Brazil, evaluated the QOL of three groups of patients undergoing cardiac surgery (pre-surgery, three and six months after surgery) found a frequency of men, being married and low schooling among the evaluated patients, similar to our findings. For smoking and diabetes association, the authors also report data similar to ours, more than half of ex-smoking and often less than one third. However, alcohol consumption was more pronounced in our research.

Regarding the score of the SF-36 among the domains, a linear increase was noted in almost all domains of the questionnaire (except for social and emotional domains), which indicates an improvement in the patients' QOL.

The research performed by Wong & Chair^[11]^, which assessed QOL after three months of heart surgery, revealed improvements in all domains of the SF-36, except for the areas of functional and social capacity.

Santana et al.^[15]^, in their research finds these different data, obtaining average of Nottingham Health Profile (NHP), a measure to assess QOL in validated chronic diseases, worsening the indicative of QOL over time after 30 and 90 days, damaging the QOL of patients undergoing cardiac surgery after hospital discharge. This difference may be due to the sensitivity of evaluation methods.

The use of models for evaluating QOL suitable for the study population should be applied specifically in the evaluation of multiple aspects of physical or mental health.

Titoto et al.^[16]^ referred to physical deconditioning due to a change in the QOL of patients undergoing cardiac surgery. It is noteworthy in this context the importance of a multidisciplinary support to these patients, especially the physiotherapist that can contribute to an abbreviation of this physical deconditioning, or the rehabilitation of these evaluated patients.

The surgical event may limit the interaction and socialization of the elderly, not to mention the limitations inherent to this age group as post-surgical complications. However, it is noteworthy that both the social field and the mental state, despite suffering reduced follow-up, still remained above 50, featuring a good perception of health in these areas.

The mental aspects can be initially affected by the disease, the adoption of "mourning", the loss of health, feeling compared to the loss of a loved one^[6]^. Thus, the health of elderly person needs comprehensive approach to be able to infer improvements in their QOL^[14]^.

The study of QOL stressed the importance of discussion of the impact of intervention strategies in cardiac surgery and health care on the QOL of elderly patients with chronic diseases.

In this research we used the SF-36, the instrument is already validated as a means of assessment of QOL through phone calls, individual interviews or in the self-administered form^[11,13]^.

In a systematic review, Jokinen et al.^[17]^ observed that the QOL tends to improve in most studies, one year after the surgical event.

Our findings indicate that the elderly population undergoing cardiovascular surgery can have benefits in improving QOL, especially in the matters: functional, physical, pain, health, vitality and emotional. Physical fitness improvement measures can be taken to resume that capability.

**Table:** 

**Authors' roles & responsibilities**	
JAFN	Analysis/interpretation of data; statistical analysis; study design; implementation of projects/experiments; manuscript writing/critical review of its content; final approval of the manuscript
LMCBR	Conception and design; manuscript writing/critical review of its content; final approval of the manuscript
MRV	Conception and design; manuscript writing/critical review of its content; final approval of the manuscript
LLCQ	Conception and design; manuscript writing/critical review of its content; final approval of the manuscript
KPLNN	Conception and design; manuscript writing/critical review of its content; final approval of the manuscript
POM	Conception and design; manuscript writing/critical review of its content; final approval of the manuscript
AFS	Analysis/interpretation of data; statistical analysis; study design; manuscript writing/critical review of its content; final approval of the manuscript
JKVRSN	Analysis/interpretation of data; statistical analysis; final approval of the manuscript; study design; implementation of projects and/or experiments; manuscript writing/critical review of its content
